# Environmental heterogeneity mediates scale-dependent declines in kelp diversity on intertidal rocky shores

**DOI:** 10.1371/journal.pone.0213191

**Published:** 2019-03-26

**Authors:** Samuel Starko, Lauren A. Bailey, Elandra Creviston, Katelyn A. James, Alison Warren, Megan K. Brophy, Andreea Danasel, Megan P. Fass, James A. Townsend, Christopher J. Neufeld

**Affiliations:** 1 Department of Botany, University of British Columbia, Vancouver, British Columbia, Canada; 2 Bamfield Marine Sciences Centre, Bamfield, British Columbia, Canada; 3 Department of Biology, University of Victoria, Victoria, British Columbia, Canada; 4 Department of Biology, University of British Columbia Okanagan, Kelowna, British Columbia, Canada; 5 Department of Biological Sciences, University of Calgary, Calgary, Alberta, Canada; 6 Department of Biology, University of British Columbia, Vancouver, British Columbia, Canada; University of Waikato, NEW ZEALAND

## Abstract

Biodiversity loss is driven by interacting factors operating at different spatial scales. Yet, there remains uncertainty as to how fine-scale environmental conditions mediate biological responses to broad-scale stressors. We surveyed intertidal rocky shore kelp beds situated across a local gradient of wave action and evaluated changes in kelp diversity and abundance after more than two decades of broad scale stressors, most notably the 2013–2016 heat wave. Across all sites, species were less abundant on average in 2017 and 2018 than during 1993–1995 but changes in kelp diversity were dependent on wave exposure, with wave exposed habitats remaining stable and wave sheltered habitats experiencing near complete losses of kelp diversity. In this way, wave exposed sites have acted as refugia, maintaining regional kelp diversity despite widespread local declines. Fucoids, seagrasses and two stress-tolerant kelp species (*Saccharina sessilis*, *Egregia menziesii*) did not decline as observed in other kelps, and the invasive species *Sargassum muticum* increased significantly at wave sheltered sites. Long-term monitoring data from a centrally-located moderate site suggest that kelp communities were negatively impacted by the recent heatwave which may have driven observed losses throughout the region. Wave-sheltered shores, which saw the largest declines, are a very common habitat type in the Northeast Pacific and may be especially sensitive to losses in kelp diversity and abundance, with potential consequences for coastal productivity. Our findings highlight the importance of fine-scale environmental heterogeneity in mediating biological responses and demonstrate how incorporating differences between habitat patches can be essential to capturing scale-dependent biodiversity loss across the landscape.

## Introduction

Ongoing biodiversity loss is expected to reduce ecosystem functioning and services [1] but uncertainty remains about the spatial scale at which to investigate the environmental drivers of such loss [2–4]. Global stressors can interact with local factors to exacerbate or ameliorate community responses to ongoing global change [5–7]. Yet, fine-scale microclimatic differences between sites are often ignored by both climate envelop models–which predict systematic shifts in the latitudinal ranges of species [8]–, and in meta-analyses of local diversity change–which group plots only by habitat-type (e.g. forest, marsh, grassland) or by region [9–11]. These common approaches, although insightful, may miss functionally important trends in community diversity change or local abundance loss if the stresses associated with a habitat patch depend more on local conditions than on regional patterns [12], or if even the most consistent declines occur at only a subset of sites within each habitat type. Understanding how to detect and predict functionally-relevant biodiversity changes will therefore depend on determining the relative importance of both broad-scale and fine-scale stressors in driving community shifts through time. While much work has focussed on how broad-scale stressors are driving the biological responses of communities [6,8,10,13], fewer studies have examined the role that local, fine-scale conditions play in magnifying or ameliorating them [6].

The rocky intertidal zone is predicted to be particularly sensitive to ongoing changes in climate [14] because intertidal organisms live near their physiological limits [5] and are sensitive to air temperatures, which tend to be more variable and extreme than seawater temperatures [5,15]. Local environmental gradients also have profound effects on intertidal systems. Environmental heterogeneity in the form of wave action plays a significant role in structuring intertidal communities [16,17]. Water movement from waves can eliminate nutrient-depleted or oxygen-rich boundary layers that are associated with low-flow environments, thereby increasing productivity [18]. Furthermore, wave splash can ameliorate the stressors associated with aerial exposure, such as desiccation and thermal stress [5,15]. Thermal profiles have suggested that wave exposed intertidal sites experience reduced thermal stress and emersion times compared to sheltered sites [15,19], suggesting that perhaps they are more resilient to rising air temperatures. Given the importance of wave action to the physiology and ecology of organisms that live along rocky shorelines, exposure to waves is likely to mediate the biological responses of intertidal organisms to ongoing changes in environmental stressors. However, the scarcity of appropriate baseline community data has made this hypothesis difficult to test in the field.

**Fig 1:**
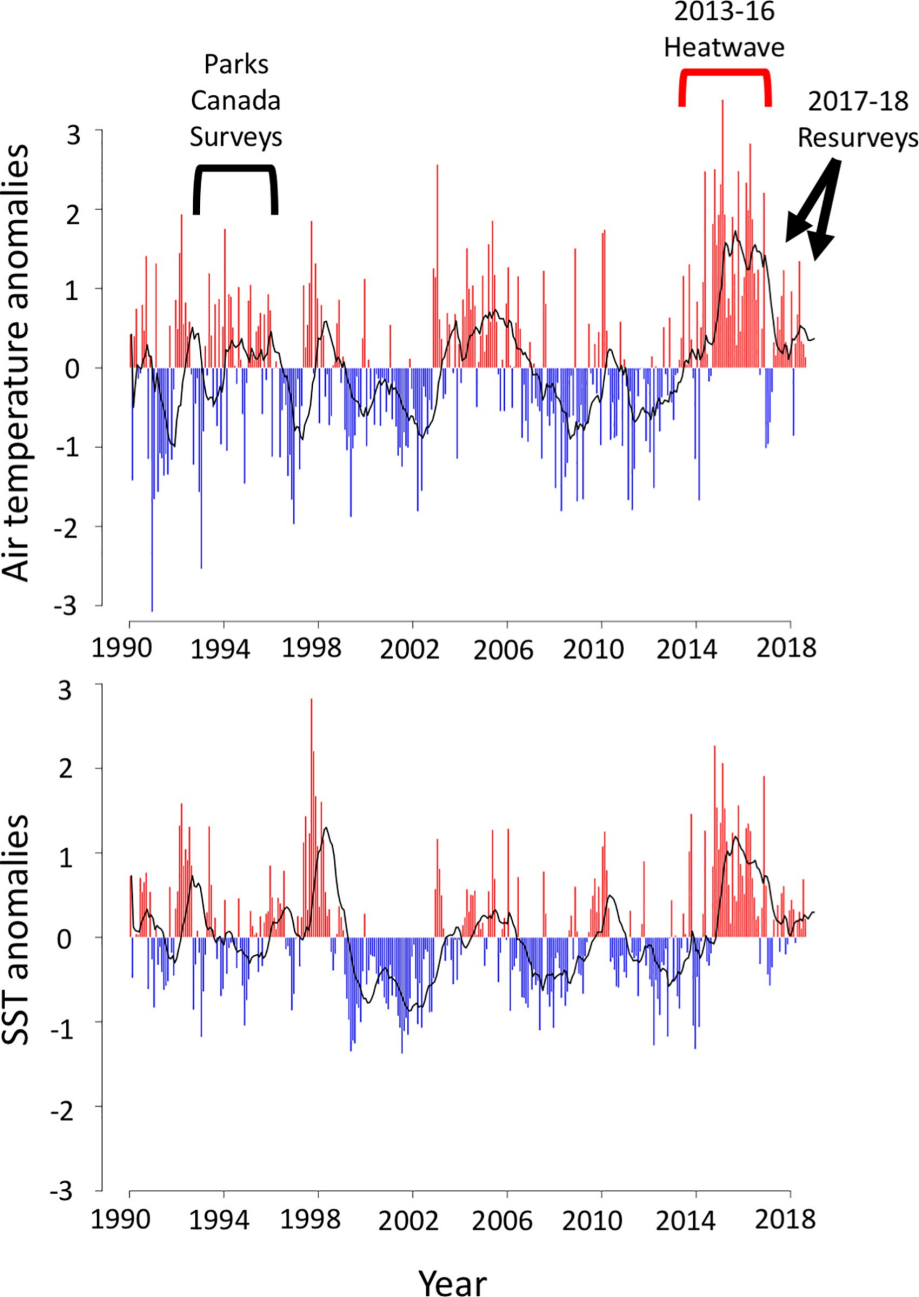
Temperature anomalies between 1990 and 2018. Data are presented for (A) air temperature and (B) sea surface temperature (SST) in relation to the timing of surveys. Data were taken from lighthouses near the opening of Barkley Sound and are calculated with respect to 33-year historical averages (dating to 1985). Also shown (as black lines) are one-year moving averages of temperature anomaly. Note that between 2014 and 2018, air temperature anomalies were consistently positive and reached an unprecedented extreme of more than 3°C in 2015. SST anomalies were also consistently positive between late 2014 and 2018.

Here we investigate the influence of a local wave exposure gradient on temporal changes in intertidal kelp bed habitats in Barkley Sound, British Columbia, Canada following 22 years of broad-scale stressors and extreme temperature events (Fig 1) [20,21]. Rocky shore kelp beds are composed of a wide variety of marine flora (e.g. seaweeds and seagrasses) and fauna (e.g. mussels, barnacles, echinoderms) many of which compete for space on the shore [22–24]. In these systems, kelps (hereafter referring only to Laminariales) act as foundation species in both intertidal and subtidal regions [22], driving ecosystem productivity through rapid growth and formation of habitat for many ecologically important species [23]. Kelps are sensitive to high temperatures [25,26], however, and as such are expected to respond negatively to climate change and climate change-amplified heat wave events [27–29]. This sensitivity to high temperatures can be made worse by the tendency for heat waves to be associated with nitrogen poor waters [30–32] that can magnify the impacts of increased temperatures [33]. Increases in marine heat wave prevalence and intensity have begun to cause negative impacts on kelp forests near their geographical range limits [13,27]. However, interactions between global, regional and local processes have led to complex responses of kelp communities, with large variability in the magnitude and direction of change [11,28,34,35]. Studies of local-scale temporal change in the abundance of kelps and other large brown algae are increasingly common [11,36–38] and have collectively demonstrated that local conditions can interact with global stressors to drive variation in ecosystem responses [11,35–37]. However, these studies have focused on a small number of species, generally in the subtidal zone, and have not examined temporal changes in the diversity or composition of entire kelp assemblages. Moreover, there is still broad uncertainty as to how natural variation in site-level environmental conditions will influence the responses of kelp-dominated ecosystems to increases in the prevalence of broad-scale stressors.

To assess temporal changes in the diversity and abundance of kelps on intertidal rocky shores, we resurveyed sites (n = 49) in 2017 and 2018 that had previously been surveyed by Druehl & Elliot between 1993 and 1995 [39]. Sites occurred broadly throughout the region, and were situated across a range of wave exposures, slopes, aspects, and types of rocky substrates. We also analysed other long-term monitoring data for one centrally located site (Wizard Islet) to better assess the timing of any widespread changes in kelp bed composition, diversity or abundance. We found substantial changes in the diversity of Barkley Sound kelp communities and widespread declines in the abundance of many kelp species. We discuss potential drivers and consequences of changes in kelp communities as they relate to gradual warming [40], the recent marine heatwave [20,21,41] and possible changes in trophic dynamics [42,43].

## Methods

### Study system

Barkley Sound, on the southwest coast of Vancouver Island, Canada, is a nearly 30 km wide inlet containing hundreds of islands. As such, it provides a wide range of local microhabitats. Both wave-sheltered and wave-exposed sites are located throughout the area both near and far from the opening to the sound. Sites were accessed by boat. Historical survey data spans 1993–1995 with most (n = 46) sites sampled twice in 1993 (n = 19) or 1994 (n = 27) and 1995. However, three sites were only sampled in either 1993 (n = 2) or 1994 (n = 1) and not in 1995 (S1 Table). Sites were located using GPS coordinates, photographs, and descriptions recorded in the original surveys. In particular, most sites were located using a photo that was often annotated with the exact location of the transect. A thorough description of most sites was also given in the original report and allowed for location of some sites that did not have photographs. Sites were only resurveyed if they could be definitively located in at least one of these two ways using distinct geographic landmarks.

### Survey techniques

Surveys were conducted following the methods of the original surveyors and were mostly restricted to species in the order Laminariales (i.e. kelps). Three non-kelp species that are expected to be more resilient to heat stress than kelps [25] were also included in the surveys but not in analyses of kelp diversity: *Phyllospadix* spp. (Alismatales), *Fucus distichus* (Fucales) and *Sargassum muticum* (Fucales). Surveys (performed between June 20 and Sept 9, 2017 and between July 10 and Aug 17, 2018) were conducted on 20–50 m stretches of coastline and included the entire intertidal region, from Lower Low Water Large Tide (LLWLT) to the upper limit of marine organisms. All sites were surveyed in 2017 and all but two (n = 47) were surveyed in 2018. A subset of these sites (N = 17) were surveyed in both June and September of 2017 and no differences in kelp community composition were detected during this time. Survey sites were uniform lengths of shoreline and included the area between the high tide line and LLWLT (approximately 3 m vertical distance). Presence and absence of all kelp species were determined for the entire survey area by carefully identifying all kelp species present by morphology. Kelps are large, seasonally persistent and are easy to distinguish based on conspicuous morphological features [39]. Thus, both our surveys and those done by the original surveyors were likely to result in unbiased, reproducible data. In order to quantify abundance, the intertidal was blocked into four zones: high intertidal (approx. > 2.5 m), mid intertidal (approx. 1.2–2.5 m), low intertidal (approx. 0.2–1.2 m) and shallow subtidal (0–0.2 m). Abundance of each species, in each zone, was then quantified based on visual estimation of percentage cover categories: absent (0%), rare (≤ 5%), common (6–20%) and (21–100%). A species’ assigned abundance was then taken from the zone of its greatest abundance.

### Wave exposure quantification

Quantifying wave exposure is a known challenge to intertidal biologists, as local topography can influence water velocities in ways that many geographical indices fail to capture [44]. For characterization of sites, we used the site-specific wave-exposure categories provided by the original Barkley Sound surveyors [39] that were modelled after the categories of Topinka *et al*. [45]. However, we grouped sites that they had ranked as “Sheltered” and “Moderately Sheltered” (into “Sheltered” grouping) since few sites were assigned to the former category. Sites were categorized by the original surveyors based on direct, qualitative observations of water motion [45] and the presence of indicator species. In order to test the validity of these categories, we measured two known proxies of wave exposure at subsets of sites. First, we used a cartographical method previously developed [46] and tested [47] in Barkley Sound. In brief, this method is a continuous index derived from the angle of unimpeded exposure to the predominant direction of offshore swell (southwest). It is therefore only effective for SW facing sites [47]. We used all of our SW (180-270^o^) facing sites (N = 26) to ground-truth these wave exposure categories (Kruskal-Wallis test: *X*^2^ = 16.451, p < 0.001; S1 Fig) and showed that sites categorized as “Sheltered” had significantly lower wave exposure index measures than “Moderate” and “Exposed” (Dunn’s test: p < 0.001 for both). There was a near significant trend suggesting a difference between “Moderate” and “Exposed” sites (Dunn’s test: p = 0.0728). Secondly, we measured the upper limit of barnacles at a majority of sites (n = 47) in each wave exposure category. This was accomplished by measuring the distance between the top of the barnacle band and the water using either a stadia rod and sight level, or—if a surface was vertical–a transect tape. Tidal predictions from the closest tide station (either Bamfield Inlet or Effingham Island) were used to calculate the height of the water relative to LLWLT for each site at the time of the survey. There was a significant effect of wave exposure category on the upper limit of barnacles, with all means differing significantly (Kruskal-Wallis test: *X*^2^ = 21.195, df = 2, p < 0.001, S2 Fig). Together, these additional measures of wave exposure suggest that our categories were appropriate.

### Shoreline classification

In order to determine how any wave exposure-specific responses might scale up across the landscape, we examined the distribution of rocky habitats of different wave exposures across the North American Pacific coast using a comprehensive georeferenced linear shoreline dataset called ShoreZone [48]. ShoreZone data span from Oregon to Alaska and are based on expert classification of shoreline units using low-elevation aerial imagery obtained from fixed-wing aircraft or helicopter and relevant geographic features. During segment classification, each shoreline unit is assigned a substrate class from high-resolution imagery, and assigned a wave-exposure class using a combination of fetch calculations and geographic and biotic features. For the current study, predominantly rocky shoreline was identified by selecting all shoreline units from this dataset which contained at least 25% rocky substrate (ShoreZone coastal classes 1–20). Regional totals of the extent of shoreline containing only bedrock (ShoreZone coastal classes 1–5) were 44–78% shorter than regional totals from mostly rocky shoreline but produced the same patterns of relative habitat types between regions. Because the average shoreline unit in the ShoreZone dataset is between 300m and 500m long (much longer than our 20–50 m surveys), the ShoreZone wave exposure classification is not able to resolve small scale differences in exposure that fall within a single shoreline unit. Importantly, ShoreZone produced similar categorizations as Druehl & Elliot when we grouped “Very Protected”, “Moderately Protected” and “Protected” Shorezone categories (hereafter “Sheltered”): 96% of sites were within one wave exposure category of one another (and classifications at 67% of sites agreed completely). Sites that differed between methods included a tidepool that was set back from the shore and protected from incoming waves, three exposed headland sites in areas that were otherwise largely sheltered from waves, and two sites that were located on the wave-sheltered side of islands that were near the mouth of the sound where overall wave exposure is greater. Barring these few exceptions that arose largely due to differences in the scale at which wave exposure was assessed, the overall concordance of the two independent approaches suggests that scaling up to the broader region using the ShoreZone dataset is appropriate.

### Long term data from Wizard Islet

In order to better evaluate the timing of any changes in kelp communities, we analyzed long-term monitoring data from a centrally-located site (Wizard Islet; 48.857983N, 125.160793W). This long-term dataset was collected by researchers at Bamfield Marine Sciences Centre and includes seven time-points between 1997 and 2017 (publically available at: https://doi.org/10.5683/SP2/C8G480). These data were collected by using randomly placed vertical transects (n ≥ 8 per year) and estimating percent cover of species in 25 cm x 25 cm quadrats at 0.5 metre tidal height increments. We summed the cover of kelps present at this site (*Egregia menziesii*, *Alaria marginata*, *Saccharina sessilis*, and *Laminaria setchellii*) and compared total kelp cover through time at tidal heights of 0.5, 1.0 and 1.5m above LLWLT. Wizard Islet was not included in the original surveys of Druehl and Elliot and so was not assigned by them to a wave exposure category. While ShoreZone classifies all of Wizard Islet as “sheltered”, we suspect that the site is actually moderately exposed given the authors’ visual observations of the site, and based on previously reported dynamometer readings [49]. While species that are indicative of wave exposed sites (e.g. *Lessoniopsis littoralis*, *Pelvetiopsis limitata*, *Postelsia palmaeformis*) are not present here, *L*. *setchellii* and *S* .*sessilis* are present, which are not generally found at sheltered sites but can be common at moderately exposed sites.

### Analysis of air and water temperature data

We analyzed air temperature and sea surface temperature (SST) from nearby lighthouses to assess whether temperature increases from gradual heating on the west coast of British Columbia [40] or persistent anomalies such as the 2013–2016 heatwave [20,21,41] have influenced thermal conditions in our study region. Air temperature data were taken from Cape Beale Lighthouse at the southern opening of Barkley Sound, and SST data were taken from Amphitrite Lighthouse at the northern opening of Barkley Sound. Air temperature data are not available from Amphitrite Lighthouse and SST data from Cape Beale have several gaps over the period of interest.

### Statistical analyses

All analyses were performed in R 3.4.1 [50]. To assess whether wave exposure and survey year significantly influenced the richness and abundance of kelps at each site, we used Kruskal-Wallis rank sum tests on site-level proportional responses (ratio of historic to modern values) with wave exposure (fixed factor, three levels) as an explanatory variable. Means were then compared using Dunn’s tests correcting for multiple comparisons. Comparisons were made to averages of historical survey data when sites were sampled in more than one year during the 1993–1995 (n = 46) or 2017–2018 (n = 47) surveys. To rule out spatial effects, we tested for spatial correlation of proportional richness responses using Moran’s I in the R package “ape” [51]. Average abundance was calculated for each site by averaging the species-specific abundance ranks of all species present. Thus, a lower value indicates a higher proportion of rare species. Rarefaction and regional species pool extrapolation were performed using the “vegan” package [52] in R and were performed for each year, combining 1993 and 1994 surveys into a single time-point. In order to determine whether individual species have changed in abundance or whether their distribution has changed across the wave exposure gradient, we used proportional odds models of abundance (ordered factor, 4 levels) versus year (fixed factor, 4 levels), wave exposure (fixed factor, 3 levels) and their interaction in the package “VGAM” [53] for the sites that were surveyed in all four years (n = 43). These models were fit to the 9 most common kelp species (*Alaria marginata*, *Costaria costata*, *Ecklonia arborea*, *Egregia menziesii*, *Laminaria setchellii*, *Lessoniopsis littoralis*, *Macrocystis pyrifera*, *Nereocystis luetkeana* and *Saccharina sessilis*) as well as *Phyllospadix* spp., *Fucus distichus* and *Sargassum muticum*. Trends in temperature anomaly from nearby lighthouses (see previous section) were assessed using a simple moving average with a 12-month window in the R package “TTR” [54]. Changes in kelp cover on Wizard Islet were assessed in two ways but in both cases statistics were performed separately for data from each tidal height (0.5m, 1.0m and 1.5m above LLWLT). First, to determine whether there were gradual changes in kelp cover through time, a linear regression was fit between annual averages of total kelp cover and year (as a continuous variable). If no linear relationship was found, then data from the most recent survey year (2017) were compared to years prior to the 2013–2016 heatwave to determine whether recent temperature anomalies [40] have influenced kelp cover. This was tested using an ANOVA and planned contrast between 2017 and the grand mean of all years sampled prior to the 2013–2016 heatwave.

## Ethics statement

Permission for sampling in the Pacific Rim National Park was granted by Parks Canada. Permission for sampling on Huu-uy-aht First Nations (HFN) territory was given by the HFN. No permission was required for sampling sites outside of these areas.

## Results

### Declines in kelp diversity and abundance were greatest at wave sheltered sites

**Fig 2:**
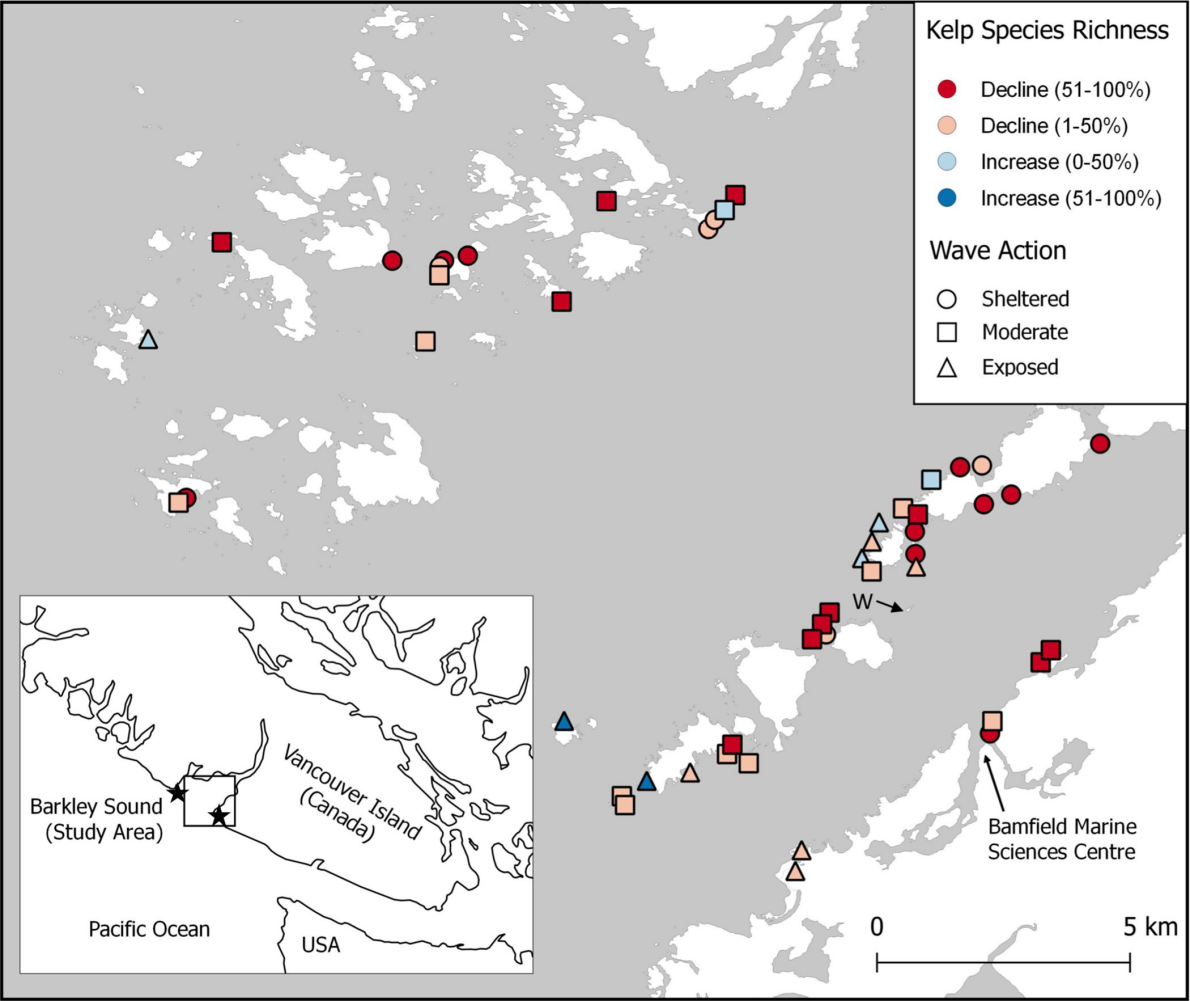
Study region. Study sites (n = 49) are coded for magnitude of change in kelp species richness (colour) and relative exposure to waves (shape). Due to the close proximity of some sites, some symbols have been moved slightly to avoid obscuring overlapping symbols. There was no effect of the spatial distribution of sites on proportional kelp richness change (Moran’s I: I = 0.0303, p = 0.49676). Stars in the inset indicate the location of Cape Beale (South) and Amphitrite (North) lighthouses. The arrow labelled “W” indicates the location of Wizard Islet long-term monitoring site.

**Fig 3:**
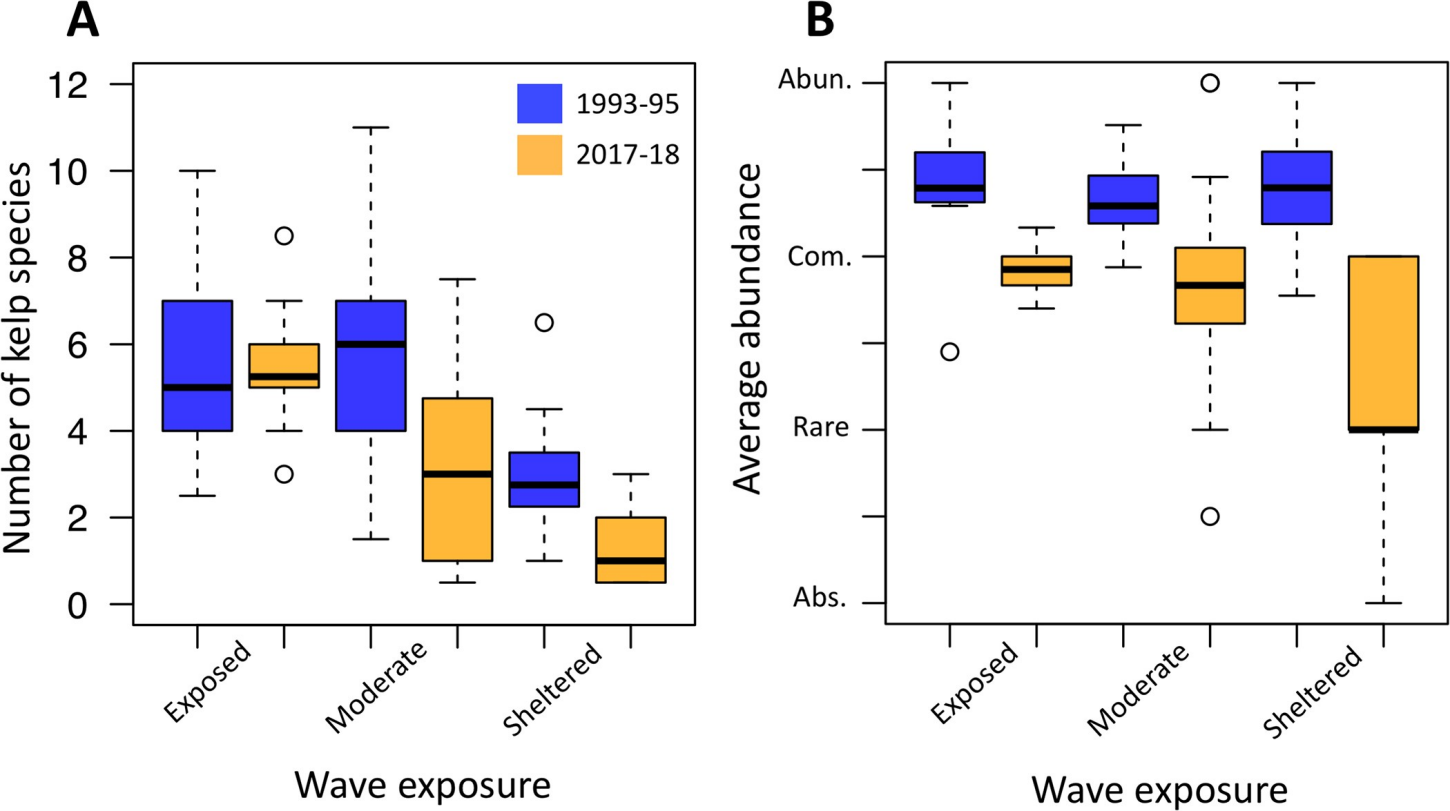
Diversity and abundance of kelp beds during 1993–1995 and 2017–2018. (A) Richness and (B) average abundance of species at each site (absent = 0, rare = 1, common = 2, abundant = 3) for historical (average of 1993–1995) and modern (2017) observations. There is a significant effect of wave exposure on site-wise proportional changes in richness (ANOVA: F_47,1_ = 17.27, P = 0.000136) and abundance (ANOVA: F_43,1_ = 4.396, P = 0.0420).

Across the study system, we found widespread declines in kelp species richness and abundance mediated by local variation in wave action (Fig 2). Kelp species richness has not changed significantly at wave-exposed sites (Paired t-test: t = 0.78779, df = 9, p = 0.4511), while kelp communities at wave sheltered sites have been reduced to between zero and three species, regardless of their historical diversity (Fig 3A), leading to a significant effect of wave exposure on proportional change in richness (Kruskal-Wallis test: *X*^2^ = 19.561, df = 2, p < 0.001, Fig 3A, S3 Fig). At all wave exposures, average abundance also declined with significantly larger declines having occurred at sheltered sites than at moderate or sheltered sites (Fig 3B, S3 Fig; Kruskal-Wallis test: *X*^2^ = 7.6663, p = 0.0216).

**Fig 4:**
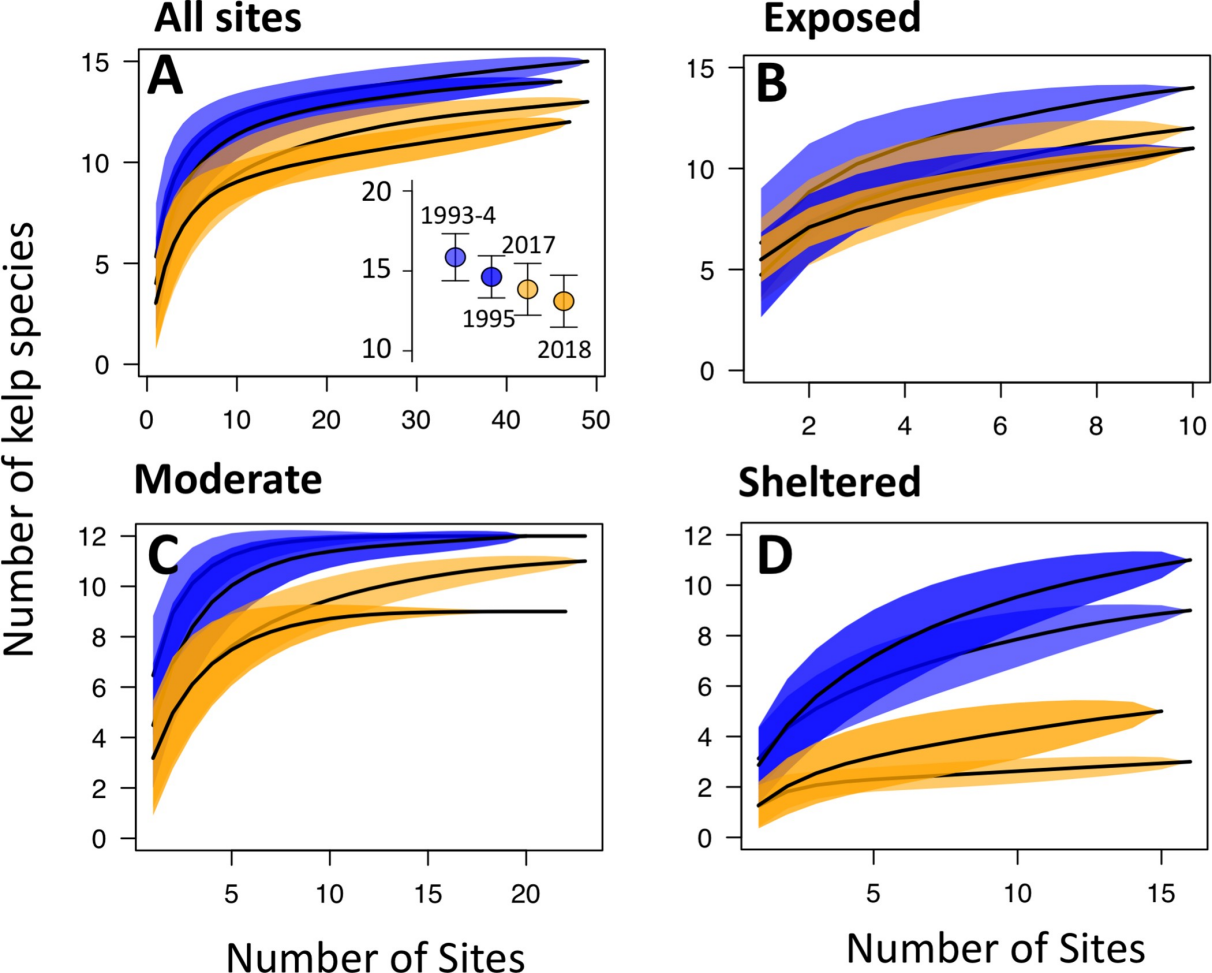
Rarefaction curves (10,000 iterations) for modern (2017) and historic (1995 for 46 sites, 1993 for 2 sites and 1994 for 1 site) surveys of sites in Barkley Sound. Analyses are broken down by (A) all sites, and then for (B) wave exposed, (C) moderately exposed sites and (D) sheltered sites plotted separately. The inset in (A) shows the results of a bootstrap extrapolation of total regional species richness of the Sound.

Habitat-dependent declines in kelp diversity were further demonstrated by changes in the shape of species accumulation curves in 2017 and 2018 relative to 1993–1995 (Fig 4). While rarefaction curves of the entire region are similar between years across all sites, sheltered sites had rarefaction curves with lower asymptotes and shallower slopes in 2017–2018 than in 1993–1995 (Fig 3), indicating that sheltered communities consisted of fewer total species during the resurveys. While at exposed sites, the total number of species detected in 2017 was slightly larger than in 1995, the total species pool at sheltered sites declined from 11 species to 3 (Fig 4; *Macrocystis pyrifera*, *Egregia menziesii*, *Alaria marginata*) with one of those species (*A*. *marginata*) only present at one site. In 2018, a total of five species were detected across all sheltered sites with only one observation of each species that was not detected at sheltered sites in 2017 (*Costaria costata* and *Saccharina latissima*).

Despite these widespread declines in the number of species found at sheltered and moderate sites between the 1993–1995 and 2017–2018 surveys, species richness across the region has not changed (Fig 4A inset, S4 Fig). Only two species were detected in 1993–1995 that were not detected during our resurveys: *Laminaria ephemera* and *Agarum fimbriatum*. These species were inconsistently found at a small number of sites during 1993–1995 and have been observed elsewhere in Barkley Sound recently. *Laminaria ephemera* was collected from Edward King Island in 2015 [55], near some of our sites, and was found in the wrack at nearby Pachena Bay (N 48.790481, W -125.120173) in 2016 (Starko, pers obs). Subtidal *Neoagarum fimbriatum* was observed reliably in Bamfield Inlet between 2012 and 2018 [55] (Starko, pers obs) and was found intertidally by three of the authors at a small island in the Broken Group Islands (N48.923916, W125.255136) in August, 2018 (Starko, Brophy & Townsend pers obs). Thus, we find no evidence that the diversity of kelps throughout all of Barkley Sound has changed, despite widespread local losses of kelp species. Our results therefore show a disconnect in how kelp diversity has changed across scales and habitats: richness of the regional kelp assemblage has remained constant, while local richness and average abundance has declined markedly, with diversity loss concentrated on wave-sheltered and moderate shores.

### Most species differed in distribution between years

**Fig 5:**
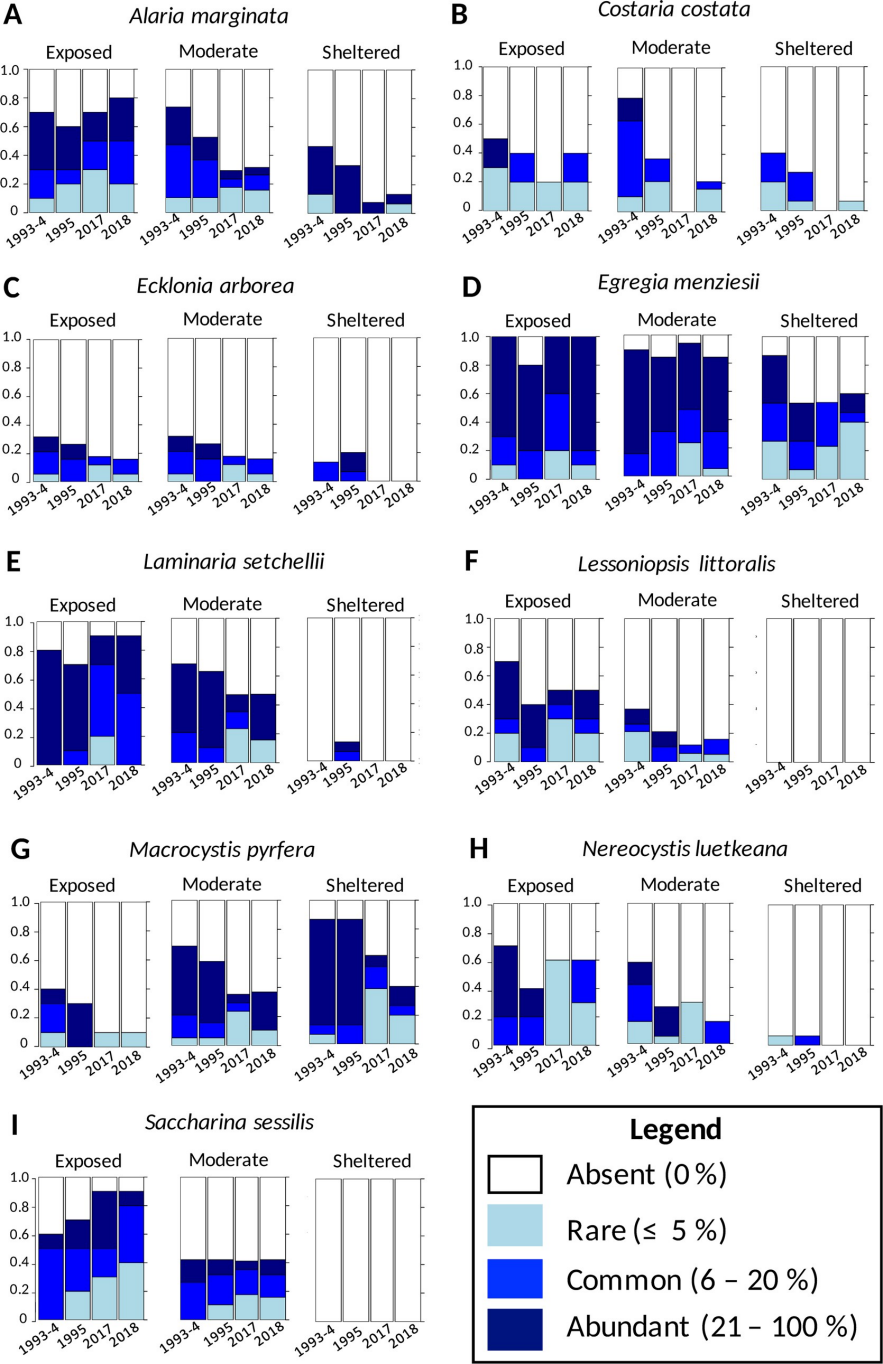
Species-specific abundance versus year for the nine most abundant kelp species at our survey sites (n = 8). Data are shown only for the 43 sites that were sampled for abundance in all four years. Data are ordinal (0 = absent, 1 = rare, 2 = common, 3 = abundant) and associated statistics are provided in Table 1.

**Fig 6:**
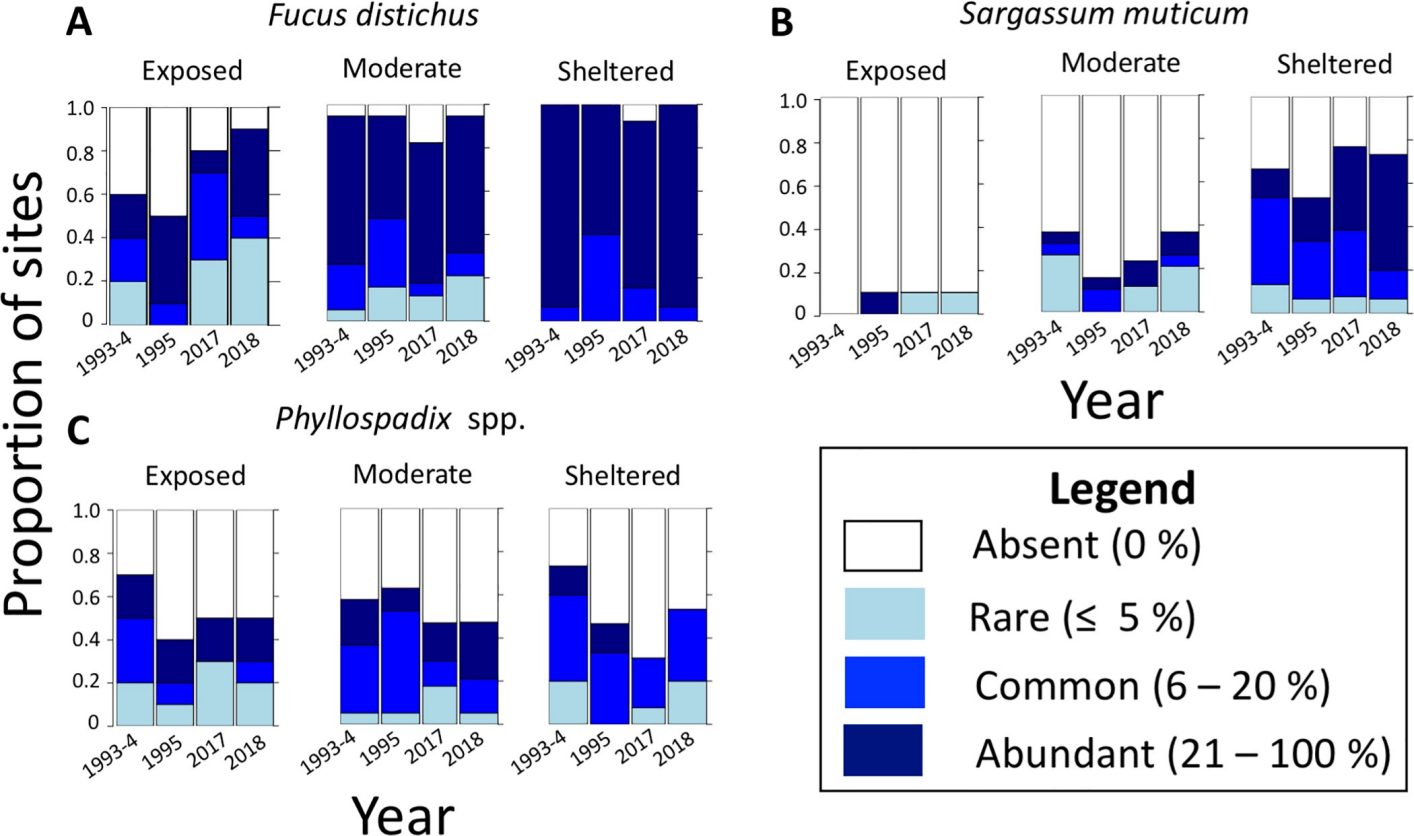
Species-specific abundance versus year for three non-kelp species (fucoids and seagrass) investigated in this study (n = 3). Data are shown only for the 44 sites that were sampled for abundance in all four years. Data are ordinal (0 = absent, 1 = rare, 2 = common, 3 = abundant) and associated statistics are provided in Table 1.

Species distributions differed between years for eight of the nine kelp species that were statistically tested using proportional odds models (Table 1; Fig 5). There was a significant effect of year on the abundance of all kelp species analyzed except for the high intertidal kelp, *Saccharina sessilis* (Table 1), and there was an effect of wave exposure on all species analyzed except *Ecklonia arborea* (Table 1). Although the abundance of *Egregia menziesii* differed between years, this was driven largely by a high abundance in 1993–1994 that was not found in 1995. When comparing data from 2017–2018 only to 1995, no significant effect of year on abundance was detected (Proportional odds model: Coefficient = -0.0444, P = 0.0828) for *E*. *menziesii*. There were particularly strong effects of exposure observed in *Lessoniopsis littoralis* and *Saccharina sessilis* which are restricted to more wave exposed sites (Fig 5). There was a significant interaction between wave exposure and year for *Alaria marginata* (P = 0.0155, Table 1, Fig 5), with a possible interaction for *Costaria costata*. (P = 0.0806, Table 1, Fig 5). These species did not decline in abundance at exposed sites but did substantially at sheltered and moderate sites (Fig 5). There was no significant effect of year or interaction between year and wave exposure for abundances of *Fucus distichus* or *Phyllospadix* spp. There was a near significant increase in the abundance of *Sargassum muticum* between 1993–1995 and 2017–2018 across all sites (Proportional odds model: Coefficient = 0.055, P = 0.0656, Table 1, Fig 6) and a significant increase across sheltered sites only (Proportional odds model: Coefficient = 1.0441, P = 0.0387). There was also a significant effect of wave exposure on abundance of *Fucus distichus* and *Sargassum muticum* across all years. This effect was particularly strong in *Sargassum muticum* that was restricted largely to sheltered areas but was found at one exposed site in both 1995 and 2017–8 (but not 1994) and was restricted to a tidepool in 2017–2018.

**Table 1:** Summary of ordinal models for 8 kelp species, 2 fucoid species and one genus of seagrass. P(*X*): P-value of the likelihood ratio *X*^2^ statistic for determining significance of each predictor.

Species	Effect	Deviance	Coefficient	P(*X*)
Kelps				
*Alaria marginata*	Exposure	16.3047	-0.138	**<0.0001**
	Year	12.8280	-86.621	**0.0003**
	Exposure*Year	5.8515	0.044	**0.0155**
*Costaria costata*	Exposure	5.3131	-0.172	**0.0212**
	Year	29.9338	-80.523	**<0.0001**
	Exposure*Year	3.0525	0.041	0.0806
*Ecklonia arborea*	Exposure	0.0168	-47.982	0.8969
	Year	6.0805	-0.094	**0.0137**
	Exposure*Year	0.7832	0.024	0.4957
*Egregia menziesii*	Exposure	31.2359	-34.381	**<0.0001**
	Year	4.7274	-0.059	**0.0297**
	Exposure*Year	1.0554	-0.018	0.3762
*Laminaria setchellii*	Exposure	71.776	-24.591	**<0.0001**
	Year	10.307	-0.024	**0.0013**
	Exposure*Year	0.241	-0.011	0.6232
*Lessoniopsis littoralis*	Exposure	44.290	-9.707	**<0.0001**
	Year	3.920	-0.050	**0.0477**
	Exposure*Year	0.038	0.006	0.8456
*Macrocystis pyrifera*	Exposure	25.467	-46.309	**<0.0001**
	Year	33.305	-0.178	**<0.0001**
	Exposure*Year	1.419	0.023	0.2335
*Nereocystis luetkeana*	Exposure	41.155	-9.107	**<0.0001**
	Year	10.021	-0.063	**0.0015**
	Exposure*Year	0.049	0.006	0.8242
*Saccharina sessilis*	Exposure	64.944	-29.045	**<0.0001**
	Year	0.042	-0.032	0.8374
	Exposure*Year	0.464	0.015	0.4957
Fucoids				
*Fucus distichus*	Exposure	37.551	-10.946	**<0.0001**
	Year	0.633	0.001	0.4262
	Exposure*Year	0.064	0.005	0.8002
*Sargassum muticum*	Exposure	51.946	34.868	**<0.0001**
	Year	3.595	0.056	0.0579
	Exposure*Year	0.517	-0.018	0.4722
				
Seagrass				
*Phyllospadix* spp.	Exposure	0.1463	-19.555	0.7021
	Year	3.6331	-0.041	0.0566
	Exposure*Year	0.3806	0.010	0.5373

### Temperature data show clear evidence of the 2013–2016 heatwave

Public data from nearby lighthouses show that both sea surface (SST) and air temperatures in Barkley Sound have reached abnormal highs between 1995 and 2017 with especially high temperatures occurring during the 2013–2016 heatwave (Fig 1). Anomalies have lasted longer and have been more extreme for air temperature than for SST (Fig 1). This heatwave is regarded as the largest on record in the north Pacific [56]. It resulted from reduced surface cooling and equatorward Eckman transport during a period of unusually high pressure in 2013–2014 (termed “the Blob”) [20,21], followed by one of the most intense El Niños on record in 2015–2016 [41]. It then dissipated by September 2016 [57]. This led to positive temperature anomalies that lasted several years (Fig 1). Although gradual heating has been documented on the west coast of Vancouver Island over longer timescales [40], there was no clear gradual temperature increase over the 22 year period of interest. However, both air temperatures and water temperatures were higher between the 5-year period of 2013–2017 than between 1991–1995 (S5 Fig) indicating that climatic conditions differed between the two survey periods.

### Declines on Wizard Islet occurred recently

**Fig 7:**
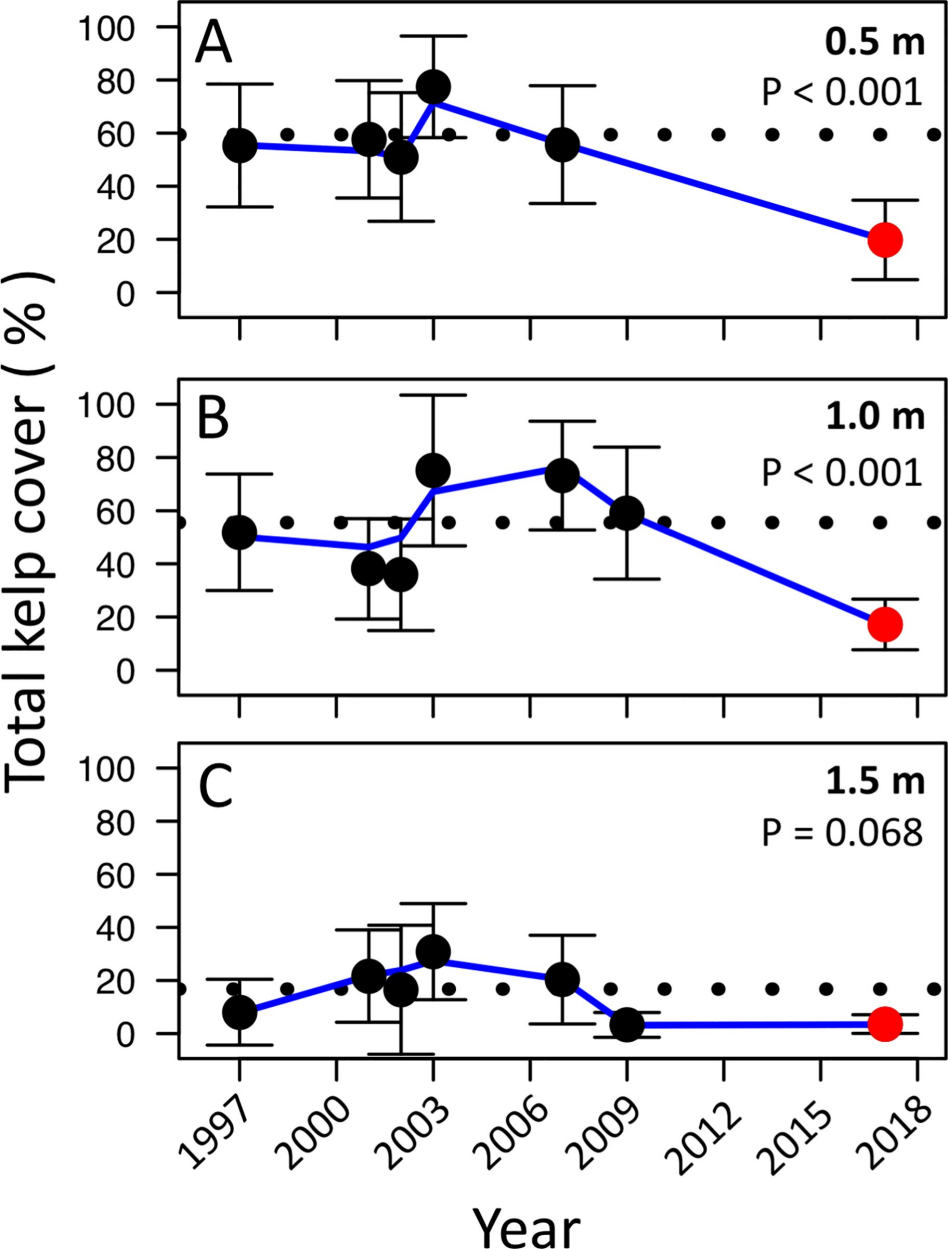
Long term trends on Wizard Islet. Total percent kelp cover through time for three tidal heights at a moderately exposed site on Wizard Islet. Data points represent average kelp cover and error bars represent 95% confidence intervals. The dotted line indicates the grand mean of kelp cover prior to the 2013–2016 heat wave. P-values indicate the significance of planned comparisons between 2017 data (mean shown in red) and all data collected prior to the 2013–2016 heat wave. There was no significant correlation between kelp cover and year, as a continuous variable (see text).

In 2017, kelp cover on Wizard Islet was lower than any other year examined (Fig 7). On average, total kelp cover was 59%, 56% and 17% at 0.5m, 1.0m and 1.5m tidal elevations respectively, prior to the 2013–2016 heatwave. In 2017, these elevations had percent cover estimates of 20%, 17% and 4%, respectively, indicating a more than two and a half-fold reduction in kelp cover at this site. Mean cover in 2017 was significantly lower than the grand mean of previous years at 0.5m and 1.0 m elevation, indicating that there was a significant drop in kelp cover at this site between 2009 and 2017 (0.5 m: ANOVA with planned comparison: F = 7.111, df = 1, p = 0.0093; 1 m: ANOVA with planned comparison: F = 14.247, df = 1, p = 0.0003). Although not significant at 1.5m elevation, there was a trend towards reduced cover in 2017 relative to the grand mean of previous years (ANOVA with planned comparison: 3.461, df = 1, p = 0.0678). There was no significant effect of year (as a continuous variable) on total kelp cover (0.5 m: Linear model: F = 4.688, df = 4, p = 0.0963; 1.0 m: Linear model: F = 0.6793, df = 5, p = 0.447; 1.5 m: Linear model: F = 1.229, df = 5, p = 0.318), likely indicating no gradual, linear decline in kelp cover through time. Thus, declines on Wizard Islet occurred between 2009 and 2017, consistent with the timing of positive temperature anomalies (Fig 1).

## Discussion

### Timing and causes of declines

Between 1993–1995 and 2017–2018 kelp beds in Barkley Sound have changed substantially with losses in kelp diversity at wave-sheltered and moderate sites. Most kelp species were found at fewer sites in 2017 and 2018 than during 1993–1995 and kelp communities in 2017–2018 tended to consist of fewer species that were less abundant on average. The spatial extent and magnitude of species loss, as well as the multiannual life cycle of many investigated kelp species suggest that these declines are a result of widespread responses to broad-scale stressors that are occurring or have occurred throughout Barkley Sound. While temperatures in Barkley Sound have gradually increased over the past century [40], this gradual change is not detectable over the 22 year period between 1995 and 2017 (Fig 1). Instead, temperature data bear a clear signal of the 2013–2016 marine heatwave, with anomalously warm temperatures detected consistently between 2013 and 2018. Thermally tolerant fucoids (*Sargassum muticum*) and seagrasses (*Phyllospadix* spp.) showed greater persistence than most kelp species, and have not declined significantly between the 1990s and 2017. Climate-mediated shifts from kelp-dominated to *Sargassum*-dominated communities have been documented elsewhere [28,29,58–61]. Therefore, these data are consistent with the hypothesis that changes in kelp communities have resulted from increases in climate stress. Data from Wizard Islet also support this hypothesis, demonstrating a substantial drop in kelp cover between 2009 and 2017, consistent with the timing of temperature anomalies. Splashing of cool water at exposed sites could alleviate air temperature stress during low tide, leading to the patterns that we show here [62] or local mixing at sites with increased water motion could mediate these stresses by preventing pockets of warm water from forming at small scales.

Out of the nine common kelp species that we investigated using proportional odds models, only two species have not declined since 1993–1995: *Saccharina sessilis* and *Egregia menziesii*. Both of these species are found higher in the intertidal zone than most other kelps, suggesting resistance to desiccation and thermal stress at low tide [39,63–65]. *Egregia menziesii* has been described as the kelp with the highest upper limit [65], although *Postelsia palmaeformis*, *Saccharina sessilis* and a wave exposed ecotype of *Alaria marginata* (i.e. *A*. *nana*) may be found as high or higher at wave exposed sites [64]. Both *S*. *sessilis* and *E*. *menziesii* possess complex three-dimensional morphologies that could promote the retention of water and may therefore improve survival during particularly stressful low tides. Although *S*. *sessilis* specializes in the high intertidal zone, it is less resistant to warm water temperatures than some species that experienced significant declines at moderate and sheltered shores (e.g. *Alaria marginata* and *Costaria costata*) [25]. This suggests that air temperature is likely a stronger driver of the observed patterns of kelp loss than SST. During the recent 2013–2014 “Blob” and the 2015–2016 El Niño, nitrogen levels were also abnormally low [38,56]. Nutrient availability may limit productivity [66] and influence thermal tolerance of kelp species [33]. So, multiple stressors could have interacted to result in the declines that we observed [33,67]. Given the multiple stressors associated with the heatwave, it is possible that different species have declined in abundance as a result of distinctive broad-scale drivers.

An alternative hypothesis, separate from the direct effects of recent temperature anomalies, is that kelp declines were caused by changes in the trophic dynamics of intertidal kelp beds. Sea stars have declined in abundance along the coast of British Columbia as a result of sea star wasting disease [43,68], an epidemic that was possibly amplified by the 2013–2016 heatwave [68,69]. This loss of sea stars has led to increases in sea urchin biomass and declines in kelp abundance in some areas [42,43]. While it is well established that herbivory by urchins can cause declines in kelp abundance, urchins are generally absent in the intertidal zone in our system, with the exception of tidepools, and therefore are not likely to be responsible for observed kelp losses. Herbivory by intertidal grazers however, especially *Katharina tunicata*, has been shown to influence kelp bed diversity and species composition in some areas [70,71]. It is unknown whether intertidal grazers are more abundant following sea star wasting disease outbreaks of 2013–2014, and it is possible that changes in trophic dynamics could have contributed to kelp losses. However, *K*. *tunicata* is predominantly found at wave exposed sites, rather than at sheltered sites [72–74], and therefore cannot have driven the ubiquity of kelp declines at moderate and sheltered sites. Declines at sheltered and moderately exposed sites occurred regardless of substratum (boulder versus bedrock) or slope (steep versus shallow), factors known to influence the distribution of invertebrates [75–77]. Therefore, the observed declines are likely too widespread to have resulted from increases in abundance of a single grazer species. Moreover, increases in grazers would have been expected to influence fucoids, such as *Sargassum*, along with kelps [78], a result which did not occur. Although we cannot rule out a role of herbivory in driving some declines, it is unlikely to be the most important driver.

Local stressors caused by human activity such as run-off or pollution are also unlikely to be drivers of the declines that we document. Barkley Sound has very low population densities, limiting human disturbance [79] and many of our sites occurred within Pacific Rim National Park, a region that is largely uninhabited and protected from human disturbance.

In sum, while changes in kelp bed composition, diversity and abundance may have resulted from multiple interacting factors, evidence is consistent with the hypothesis that temperature anomalies during the 2013–2016 heatwave drove widespread declines in kelp bed diversity and species abundance. Regardless of the timescale over which these declines occurred or the exact combination of factors that have driven them, our results suggest that wave-sheltered habitats are more sensitive to regional stressors than wave exposed habitats.

### Implications of kelp loss

**Fig 8:**
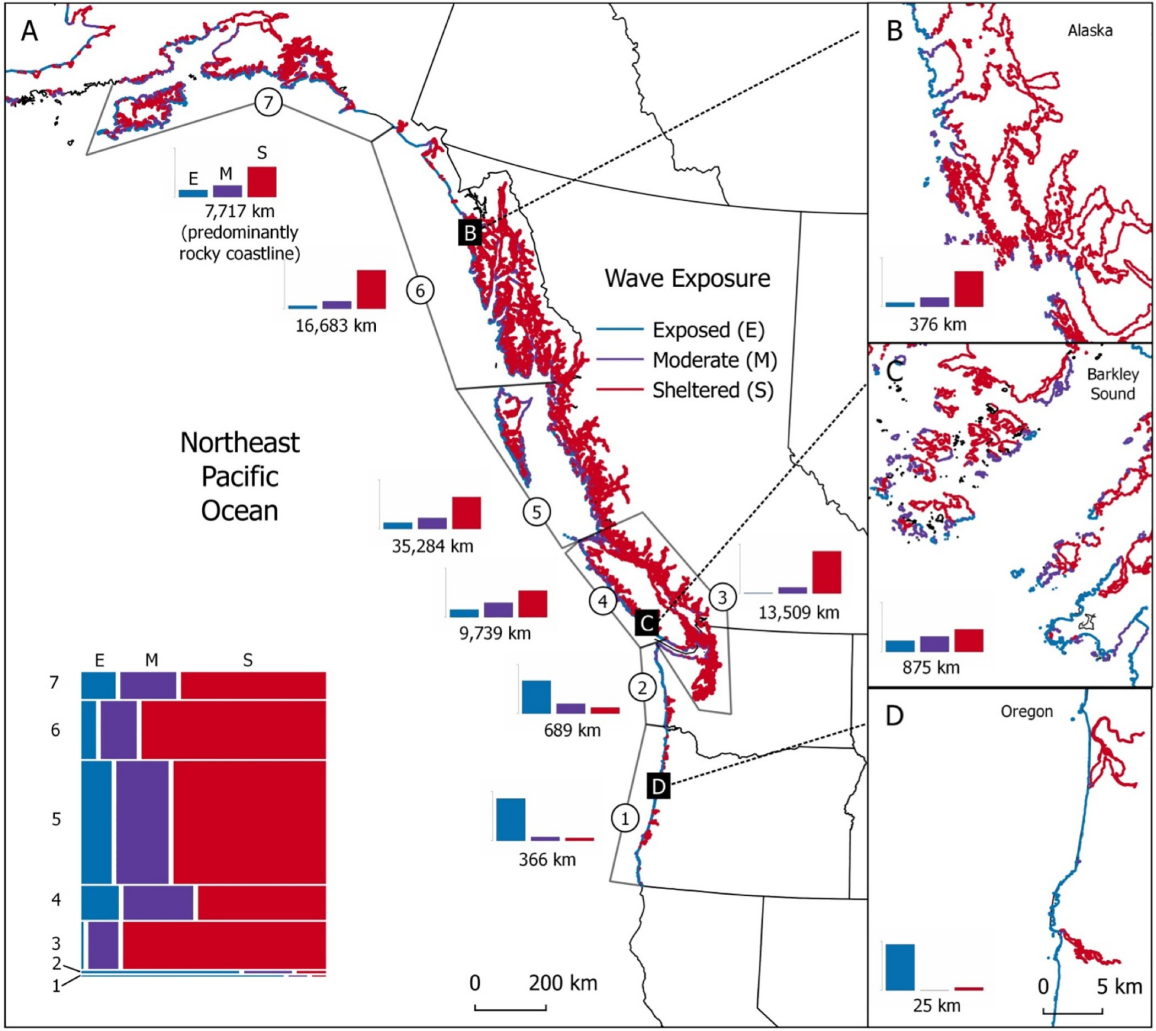
Northeast Pacific intertidal habitat classified by wave exposure. Wave-sheltered habitat makes up the majority of Northeast Pacific shorelines (A) and is abundant in British Columbia (C) and Alaska (B) but rare along the outer coast of Washington and Oregon (D). Bar plots show the proportions of rocky shoreline of different wave-exposures and are accompanied by the length of predominantly rocky shoreline in each region (A) or in each inset (B-D). Mosaic plot inset in A shows the relative proportions of rocky shoreline of different wave-exposures scaled by the length of coastline for each region. Regions from South to North: (1) Oregon coast, (2) Washington outer coast, (3) Salish Sea, Puget Sound, and Strait of Georgia, (4) western Vancouver Island, (5) northern British Columbia, (6) southern Alaska, (7) central Alaska.

Given the important ecological role of kelp [22,23,80], the substantial declines that we document are likely to have cascading effects on the diversity of other organisms and on ecosystem functioning and productivity of intertidal communities [22]. While the affected kelp communities may yet recover following the 2013–2016 heatwave, our results offer a novel prediction for how communities will be affected by increasing climatic stressors. In particular, these results suggest that kelp communities at wave-sheltered sites may be particularly sensitive to the increasing prevalence of broad-scale stressors, such as more frequent and intense marine heat waves [81–84]. It could be hypothesized that declines on wave-sheltered shores may not affect regional productivity or habitat availability as much as would declines on wave-exposed shorelines, which are more diverse and more productive [17]. Yet, positive interactions generated by kelp canopies may be especially important on wave-sheltered shores because these shores are more physiologically stressful [85]. Furthermore, the lower diversity and productivity of sheltered shorelines is far outweighed by their sheer abundance in the Northeast Pacific (Fig 8).

Approximately 57,000 km of wave-sheltered rocky shoreline exists from Oregon to central Alaska, virtually all of which (99.8%) occurs north (and east) of Washington’s outer coast (Fig 8A). Therefore, even small changes in kelp diversity on more sensitive wave-sheltered shores could have large effects on intertidal productivity if magnified across the landscape. While some of this shoreline may not be suitable kelp habitat due to limitations from salinity and other factors, it is clear from our analyses that wave sheltered shorelines are common and extensive in northern Washington, British Columbia and Alaska. Given that these types of habitats are uncommon further south, it is likely that some northern shorelines will experience losses in kelp abundance and diversity before southern ones.

The sensitivity of wave-sheltered sites in our system is contrasted by the apparent resilience of wave exposed kelp beds, a novel finding that has important implications for conservation and management. Wave exposed sites are highly productive and often represent hotspots of diversity in our system [39]. Our results demonstrate that these sites may also be especially resilient against broad-scale stressors. Wave exposed sites may act as refugia during times of stress, potentially buffering kelp ecosystems against regional extinctions and playing a key role in maintaining regional species diversity. The role of climatic refuges in maintaining species diversity through geological time has been widely discussed in the paleoecological literature [86,87]. However, few studies illustrate this phenomenon under ongoing global change.

Recent efforts to understand biodiversity change in ecologically important biogenic habitats have identified areas where ecosystems are performing substantially better (“bright spots”) or worse (“dark spots”) than average [88]. Our results demonstrate that a fine-scale environmental gradient–one that can vary over tens of metres [44]–has mediated the formation of bright spots and dark spots in kelp-dominated ecosystems. Importantly, given the distribution of wave exposure in the Northeast Pacific (Fig 8), such dark spots are likely to be much more common than bright spots across the landscape. As a result, although wave exposed sites might maintain regional diversity, abundant dark spots could have profound effects on ecosystem functioning and coastal productivity [22,24,89].

In addition to reductions in diversity, we document widespread declines in the abundance of intertidal kelps in Barkley Sound. While the magnitude of decline was dependent on wave exposure (Fig 3B) and varied between species (Figs 6 and 7), sites from all wave exposure categories declined significantly in average kelp abundance and 7 of the 9 species most common species declined in abundance. Losses of kelp cover are common worldwide [29] and a recent global meta-analysis found that more than one third of published subtidal kelp bed surveys showed declines over the past 50 years–significantly more than had increased [11]. While many negatively affected kelp forest ecosystems are found near the warm-edge of kelps’ latitudinal range [13,28,34,90], our data suggest that similar declines have occurred in the intertidal zones of British Columbia, reasonably far from the warmer latitudinal limit of northeast Pacific kelp ecosystems [91]. This supports previous work suggesting that central-and not just edge- populations of brown algae may be susceptible to broad-scale stressors brought on by heat waves [37,92]. This may be especially true for intertidal communities that show limited correlations between latitude and thermal stress [5].

Although declines may be attributable to stressors occurring over short timescales [56] (Fig 1), rather than a response to gradual warming, the recovery from ecosystem-wide declines may not occur rapidly in either case. Four of our sites lost all kelp species and thirteen others were reduced to a single, sometimes rare (< 5% cover) species. Thus, many of our sites have experienced complete or near-complete collapses of kelp-dominated communities. For the 17 sites that had the fewest kelps in 2017, similar results were found in 2018: four sites with no kelps and 13 sites with only one kelp species (data in S1 and S2 Tables). Thus, even if declines did occur recently, they have persisted for two years, indicating that recovery has not occurred immediately following the heatwave. Kelp bed collapses have been documented previously in various regions worldwide and many have yet to recover following initial kelp bed collapse [28,93,94].

### Scale-dependence of diversity loss and the importance of local gradients

A broader implication of our results, one that extends beyond rocky shores, is that important biodiversity loss could easily remain hidden from studies not specifically designed with environmental heterogeneity in mind. We found that the total diversity of kelps in Barkley Sound has not changed throughout the region, yet a majority of sites experienced large losses in local diversity. This clearly demonstrates how declines in diversity can be concentrated in only some habitats that may be stressful and lower diversity to begin with. Studies that focus on regional patterns or only investigate certain types of sites could miss losses mediated by local gradients. Thus, differences between local conditions in distinct habitat patches may directly contribute to the disconnect between diversity measurements taken at different spatial scales [1,89]. In our study, species accumulation curves demonstrate how we could have missed the widespread biotic homogenization that has occurred only at wave-sheltered sites were we to assess all sites together (Fig 5). Capturing these losses in between-site diversity can be essential to monitoring and conservation efforts because ecosystem functionality can depend on having many species combinations across the landscape [1,95]. Yet, while our results support growing evidence that local environmental heterogeneity explains important variation in diversity loss [7,14], few studies that examine responses to ongoing global change incorporate these gradients into their analyses. Our results point to the need for a framework that better incorporates the interacting effects of stressors at different scales. Such an approach would hold much promise for identifying and predicting diversity loss and changes in abundance not only at species range edges but also along local gradients throughout the range of each species.

Heterogeneity in environmental variables, like wave exposure, is ubiquitous in the natural world, but its importance in determining the responses of communities to broad scale stressors is often underappreciated [6]. As global change continues to drive shifts in ecosystem structure, heterogeneity of habitats will lead to variation in microclimates [5,19] and could strongly affect the biological responses of organisms. Rather than assessing the average responses across all communities in a region or across the globe [9,10], we should work to identify the habitats that are most vulnerable to declines and determine whether they are abundant enough to influence ecosystem functioning across the landscape. Consistent declines across all habitat patches or at the most diverse, high quality habitats may not be reasonable predictions for how communities will respond to global change [96]. Instead, some sites may act as refugia, while diversity is lost from marginal habitats; if these sensitive habitats are common, as they are in our system, then the consequences to ecosystem functioning could be profound.

## Supporting information

S1 Table Summary of survey data for all sites sampled in this study. (CSV)

S2 Table Raw data for communities surveyed in this study. Excel spreadsheet with abundance and presence/absence data for all five survey years.
(XLSX)

S1 Fig Boxplot of cartographical Wave Exposure Index measure versus the wave exposure categories used in this study, for SW facing sites (n = 26). Letters represent significant differences between means as determined by a Kruskal-Wallis rank sum test followed by a Dunn’s posthoc test.
(TIFF)

S2 Fig Boxplot of the upper limit of barnacles at sites (n = 47) of each wave exposure category. Letters represent significant differences between means as determined by a Kruskal-Wallis rank sum test followed by a Dunn’s posthoc test.
(TIFF)

S3 Fig **Proportional, site-level changes in (A) richness and (B) average abundance, broken down by wave exposure.** Both panels display ratios of modern and average historic observations and red lines indicate zero change.
(TIFF)

S4 Fig Results of species pool bootstrap extrapolation between years. Estimates shown for (A) all sites, (B) exposed, (C) moderate and (D) sheltered species pools, broken down by year, as calculated using the specpool function in the R package ‘vegan’. Points represent bootstrapped estimates of species richness and error bars represent 95% confidence intervals.
(TIFF)

S5 Fig Maximum air and water temperatures between the periods of 1991–1995 and 2013–2017. Panel (A) shows the maximum daily air temperature averaged by month and by time-period; data is from Cape Beale Lighthouse. Panel (B) shows the maximum monthly sea surface temperature averaged by time-period; data is from Amphitrite Lighthouse. Both lighthouses are located on the outer edge of Barkley Sound, British Columbia.
(TIFF)
