# Supplementary figures and images for: Environmental heterogeneity mediates scale-dependent declines in kelp diversity on intertidal rocky shores
Source: PLoS One. 2019 Mar 26;14(3):e0213191. doi: 10.1371/journal.pone.0213191 (PMC6435185; doi:10.1371/journal.pone.0213191)

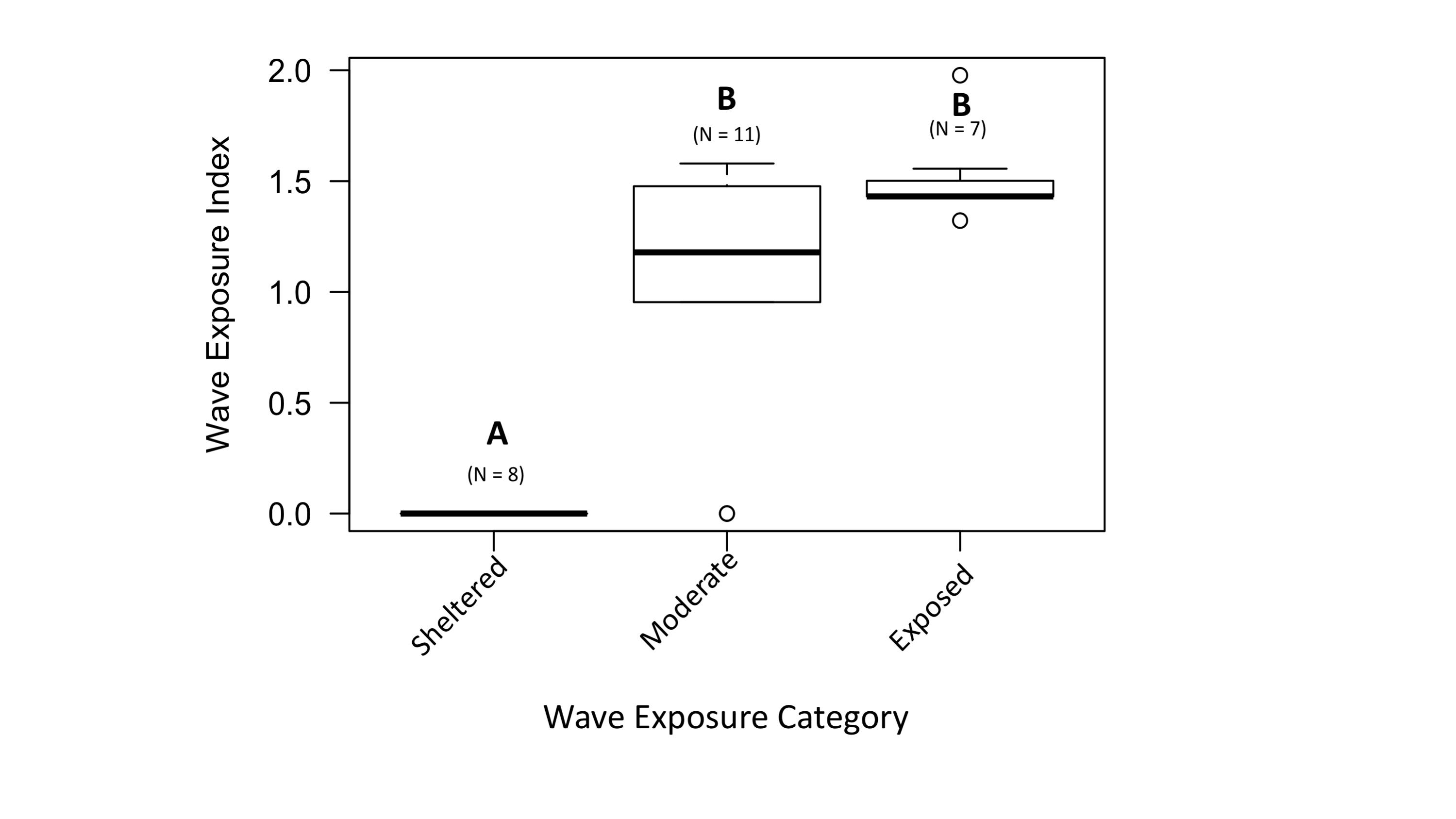

Supplement: S1 Fig — Letters represent significant differences between means as determined by a Kruskal-Wallis rank sum test followed by a Dunn’s posthoc test. (TIFF) [file pone.0213191.s003.tiff]

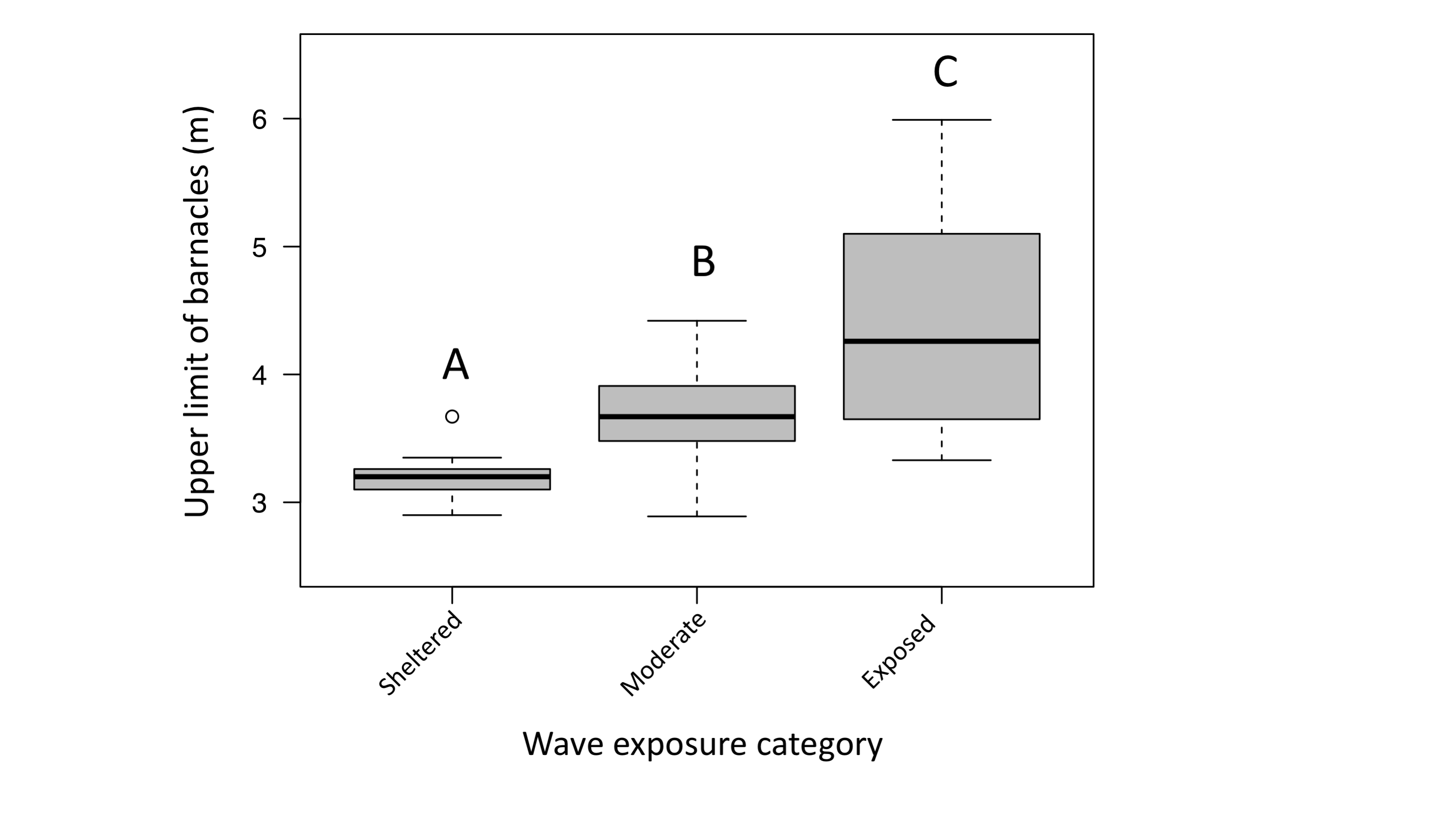

Supplement: S2 Fig — Letters represent significant differences between means as determined by a Kruskal-Wallis rank sum test followed by a Dunn’s posthoc test. (TIFF) [file pone.0213191.s004.tiff]

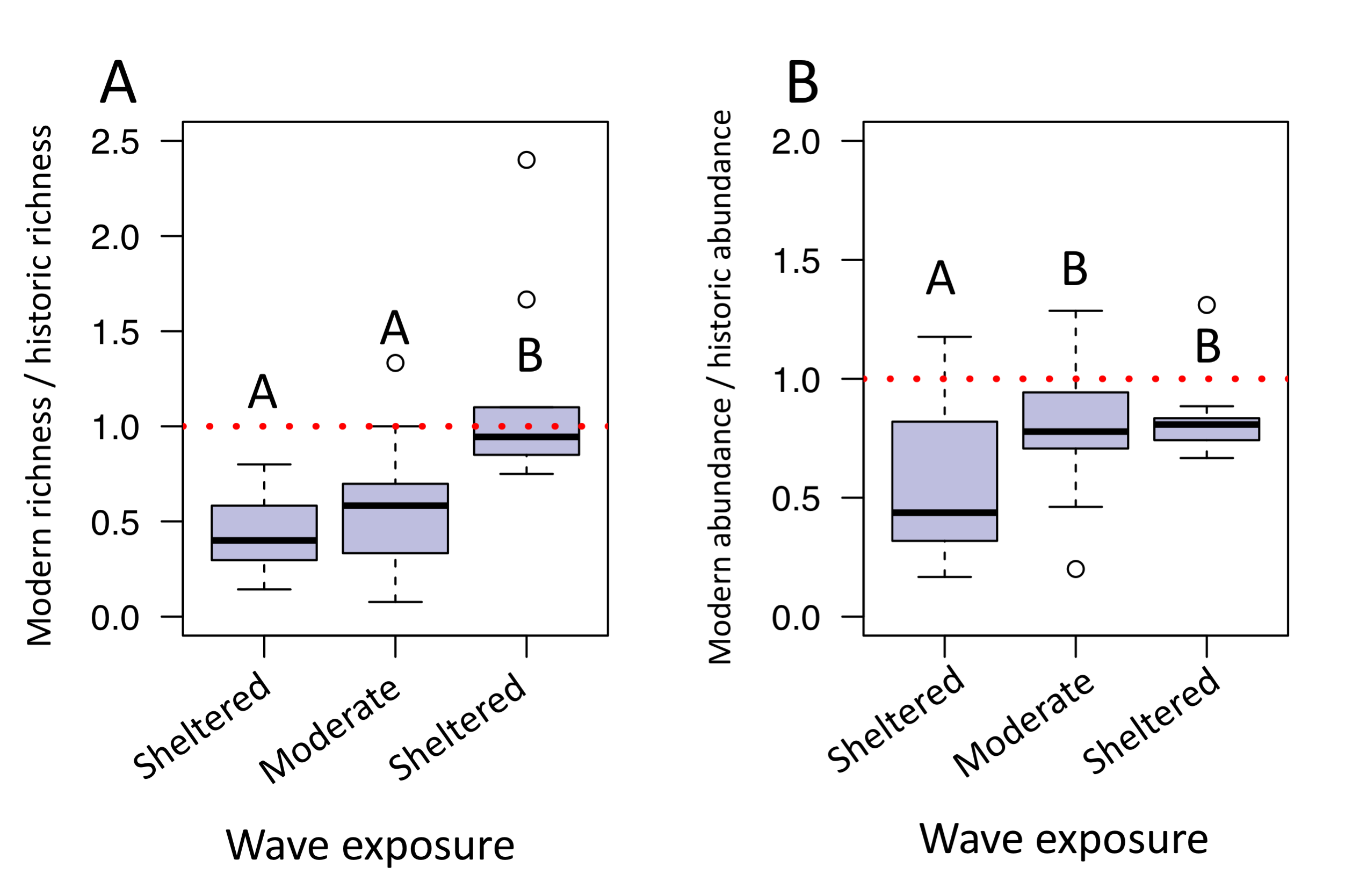

Supplement: S3 Fig — Proportional, site-level changes in (A) richness and (B) average abundance, broken down by wave exposure. Both panels display ratios of modern and average historic observations and red lines indicate zero change. (TIFF) [file pone.0213191.s005.tiff]

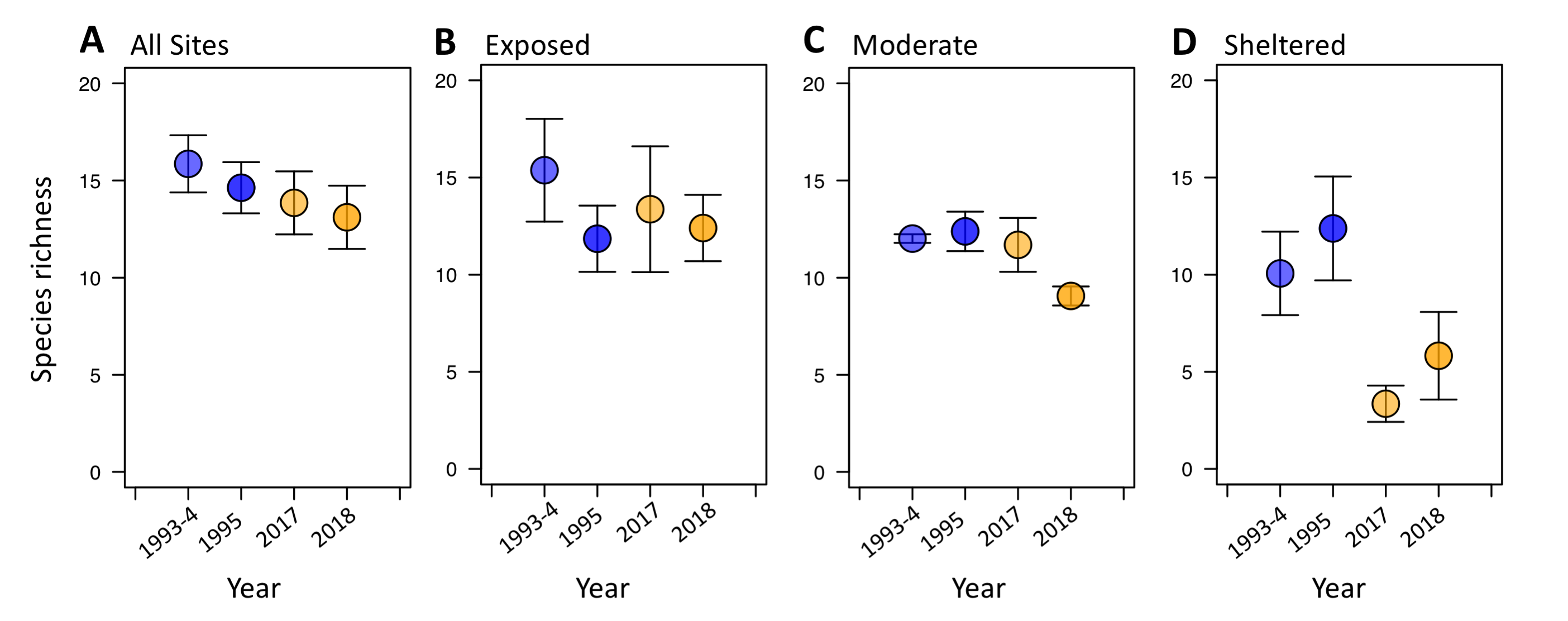

Supplement: S4 Fig — Estimates shown for (A) all sites, (B) exposed, (C) moderate and (D) sheltered species pools, broken down by year, as calculated using the specpool function in the R package ‘vegan’. Points represent bootstrapped estimates of species richness and error bars represent 95% confidence intervals. (TIFF) [file pone.0213191.s006.tiff]

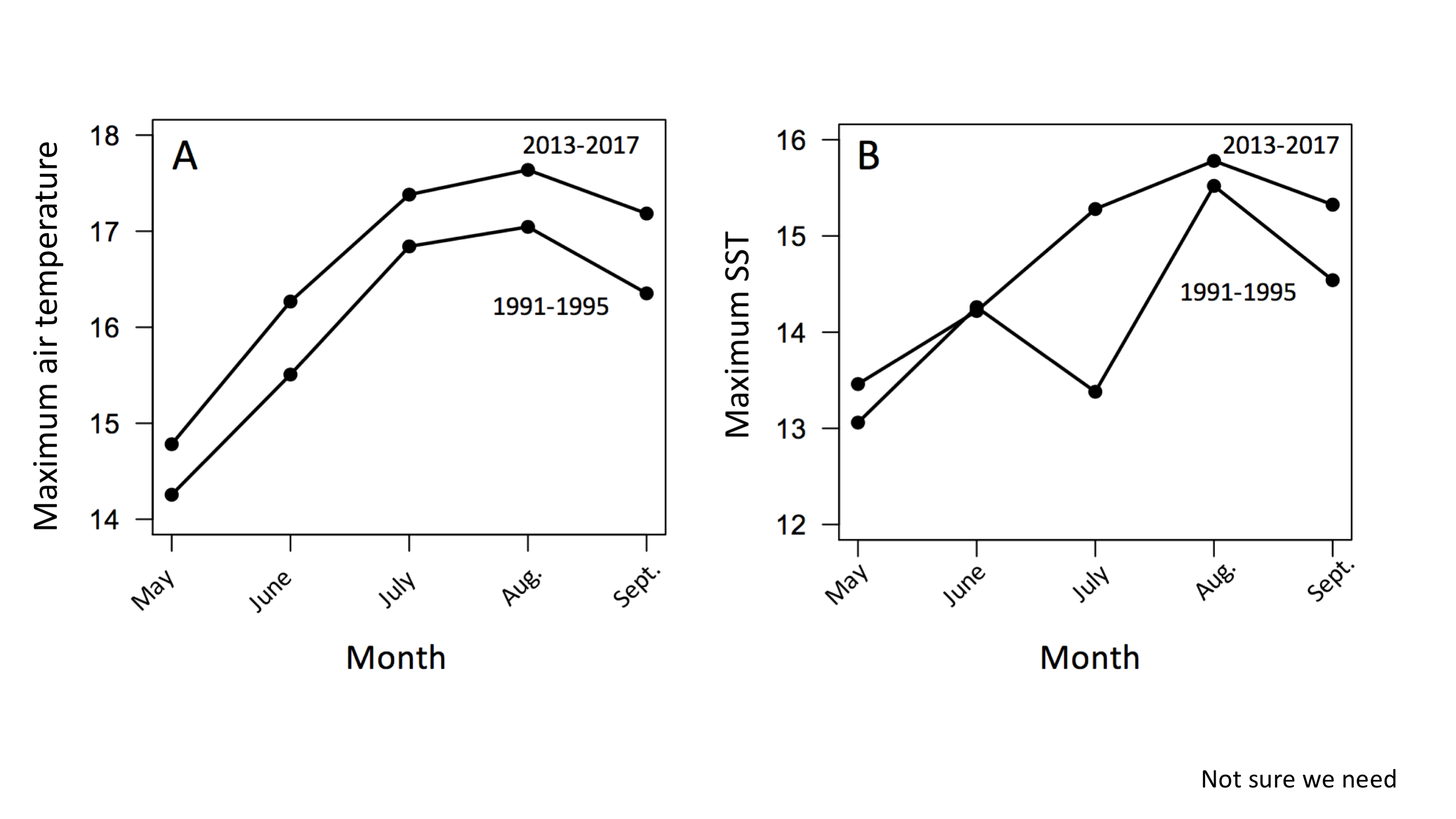

Supplement: S5 Fig — Panel (A) shows the maximum daily air temperature averaged by month and by time-period; data is from Cape Beale Lighthouse. Panel (B) shows the maximum monthly sea surface temperature averaged by time-period; data is from Amphitrite Lighthouse. Both lighthouses are located on the outer edge of Barkley Sound, British Columbia. (TIFF) [file pone.0213191.s007.tiff]
